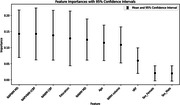# MRI Correlates of Executive Function in Cognitively Normal Older Subjects

**DOI:** 10.1002/alz.093846

**Published:** 2025-01-09

**Authors:** Banafsheh Shakibajahromi, Sudipto Dolui, Christopher Brown, Mohammad Taghvaei, Pulkit Khandelwal, Qichao Wu, Shokufeh Sadaghiani, Paul A. Yushkevich, David A Wolk, John A. Detre

**Affiliations:** ^1^ Department of Neurology, University of Pennsylvania, Philadelphia, PA USA; ^2^ Department of Radiology, University of Pennsylvania, Philadelphia, PA USA; ^3^ University of Pennsylvania, Philadelphia, PA USA; ^4^ Penn Image Computing and Science Laboratory (PICSL), University of Pennsylvania, Philadelphia, PA USA

## Abstract

**Background:**

Cerebral small vessel disease (CSVD) is the most prevalent cause of vascular cognitive impairment. Executive function impairment and white matter (WM) lesions occur early in CSVD, the latter typically in periventricular WM (PVWM) ‐ the least perfused brain region. Accounting for vascular risk factors (VRF) and amyloid status in cognitively normal older subjects, we assessed the relationship between Trail‐making test (TMT) performance and MRI measures preceding WM lesions: arterial spin labeling (ASL)‐derived cerebral blood flow (CBF) and diffusion tensor imaging (DTI) indices in normal‐appearing WM (NAWM) and normal‐appearing PVWM (NAPVWM).

**Method:**

133 cognitively normal adults (>50y) from Penn Alzheimer’s Disease Research Center were included. TMT:B minus TMT:A (TMT:B‐A) was used as a metric of executive function. We categorized participants into amyloid‐positive (A+) and amyloid‐negative (A‐) groups based on PET. VRFs included hypertension, diabetes, obesity, hypercholesteremia and smoking. WM hyperintensity (WMH) masks were derived from FLAIR MRI using a deep‐learning model to calculate WMH volume and to mask WMH voxels. We extracted mean CBF and DTI indices (fractional anisotropy (FA) and mean diffusivity (MD)) in NAWM and NAPVWM using a perfusion‐based PVWM mask. NAPVWM relative CBF (rCBF) was defined relative to global CBF. Using linear regression, we assessed the associations of TMT:B‐A with VRF presence and imaging metrics in each group adjusting for age, sex and education. The relative importance of these variables was measured through random forest regression.

**Result:**

The mean (SD) age was 70 (6) with 64% females. 23 subjects were A+ and 79 were A‐. Neither VRF presence nor imaging metrics were significantly associated with TMT:B‐A in A+ group. In A‐ group, after adjustment for covariates, VRF (Beta= 0.319, P=0.009), NAPVWM rCBF (Beta= ‐0.240, P=0.035)), NAWM MD (Beta=0.266, P=0.022) and NAPWM MD (Beta=0.255, P=0.029) were significantly associated with TMT:B‐A. WMH volume was not associated with TMT:B‐A (P= 0.668). In A‐ group, NAPWM MD and NAPWM rCBF were the most important determinants of TMT:B‐A relative to the other variables in the random forest model (Figure 1).

**Conclusion:**

CBF and MD in NAPVWM are associated with executive function in normal aging and might be an early biomarker of CSVD.